# Point-of-care *BCR::ABL1* transcript monitoring using capillary dried blood in chronic myeloid leukemia patients

**DOI:** 10.1038/s41375-024-02285-9

**Published:** 2024-06-15

**Authors:** Olga Sala-Torra, Lan Beppu, Qian Wu, Emily Welch, Erwin Berthier, Jerald P. Radich, Vivian G. Oehler

**Affiliations:** 1 Translational Science and Therapeutics Division, Fred Hutchinson Cancer Center, Seattle, WA USA; 2 Clinical Research Division, Fred Hutchinson Cancer Center, Seattle, WA USA; 3 Tasso, Inc., Seattle, WA USA; 4 Department of Medicine, Division of Hematology and Oncology, University of Washington, Seattle, WA USA

**Keywords:** Chronic myeloid leukaemia, Preclinical research

## To the Editor:

Chronic myeloid leukemia (CML) comprises ~15 to 20% of all adult leukemias and the development of oral BCR::ABL1-targeted tyrosine kinase inhibitors (TKIs) has revolutionized management. For most chronic phase (CP) CML patients age-matched survival is similar to the general population [1]. The consequence of this greatly improved overall survival (OS) is that the number of individuals living with CML and requiring continued treatment and disease monitoring is steadily increasing. Adherence to treatment and regular monitoring of treatment responses are crucial to achieve long-term survival as specific treatment response milestones map to risk for disease progression. In the absence of effective therapy, the risk for CML progression to an acute leukemia with poor prognosis remains. Response to therapy is typically measured in the peripheral blood by quantitative reverse-transcription polymerase chain reaction (RT-PCR). Specific *BCR::ABL1* transcript levels, reported as percentages relative to a control gene (e.g., *ABL1*) and standardized on the International Scale (IS), such as *BCR::ABL1 ≤* 1% are associated with improved OS. Deeper molecular responses such as *BCR::ABL1 ≤* 0.1%, also called major molecular response (MMR), limit the likelihood of losing response. The US National Comprehensive Cancer Network and European LeukemiaNet recommend monitoring every 3–6 months to confirm adherence to therapy and ensure treatment milestones are met and maintained [2, 3]. Molecular response also dictates when treatment changes should be considered, and delays can result in disease progression.

An analysis of 1188 newly diagnosed CML patients on Medicare in the US highlighted that only 32% had optimal monitoring defined as at least three tests in the first year [4]. For patients living in remote geographic locations, monitoring can be financially burdensome due to the need for time off from work and the cost of travel. This study also identified disparities in testing based on race, ethnicity, and socioeconomic status. The gap between recommended and actual testing is even more profound in low- and middle-income countries [5]. In contrast to Europe and North America, the median age of CML diagnosis is substantially younger in other regions and a recent forecast model estimated that 53% of individuals with CML are living in resource-limited settings [6]. An investigation of the health burden of CML globally identified that the age-standardized incidence rate of CML is increasing in Central Sub-Saharan Africa, Andean Latin America, and Southeast Asia [7]. Moreover, Central Sub-Saharan Africa also showed an upward trend of age-standardized death rate and disability-adjusted life years. Consequently, the need for low-cost point-of-care (POC) assays that facilitate patient-directed blood collection for CML diagnosis and monitoring is high because effective therapies are available through pharmaceutical company programs and organizations like the Max Foundation (Seattle, WA) [8].

**Fig. 1:**
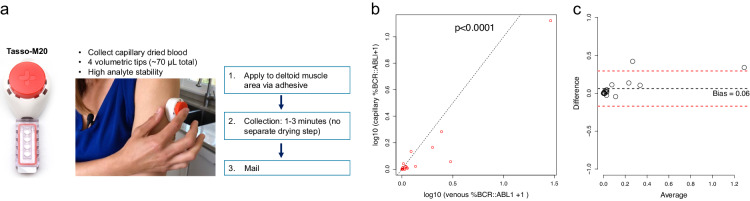
Capillary dried blood sampling by Tasso-M20 and correlation of venous vs. dried capillary *BCR::ABL1%.* **a** Overview of the Tasso-M20 device. **b** After log_10_ transformation, R is 0.99 between the two raw measures and 0.96 between the two log_10_ transformed measures (log_10_ (*x* + 1) vs. log_10_ (*y* + 1)). **c** Bland-Altman Plot showing the bias as the difference between the log_10_ transformed values of the venous and capillary samples (log_10_ (*x* + 1) and log_10_ (*y* + 1)). Bias = 0.06.

To address this gap, we have developed and tested technologies to facilitate POC monitoring. Dried blood spots (DBS) have a long history of use [9]. DBS are low cost and have limited storage needs and a low infection hazard. In earlier published work, we demonstrated that DBS (venous blood on filter paper) were stable for approximately 43 days, and that DBS *BCR::ABL1* transcripts correlated highly with venous *BCR::ABL1* transcripts as measured using GeneXpert (Cepheid, Sunnyvale, CA, USA), a machine used routinely in clinical practice [10]. Patient-directed POC collection of dried capillary blood (DCB) using Tasso-M20 (Tasso, Inc., Seattle, WA, USA) avoids venipuncture and the need to travel to a laboratory or clinic. The device collects volumetrically controlled dried blood samples (Fig. 1a). A sample can be collected at home and mailed to a regional laboratory without any additional transport requirements. This approach could increase both diagnostic and monitoring access for individuals living remotely or with limited access to healthcare.

To establish the sensitivity of DCB for e13a2 and e14a2 *BCR::ABL1* transcript monitoring we compared results collected using the Tasso-M20 device to routine standard of care *BCR::ABL1* transcript monitoring performed on venous blood at a CLIA certified clinical lab at Fred Hutchinson Cancer Center (Fred Hutch). Twenty patients were enrolled on Fred Hutch protocol RG1004335. The study was performed in accordance with the Declaration of Helsinki and informed consent was obtained. Clinical *BCR::ABL1* transcript monitoring was performed using Xpert® BCR-ABL Ultra (Cepheid), which requires 4 ml of venous blood. The assay detects *BCR::ABL1* transcript types e13a2 and/or e14a2 and results are expressed as *BCR::ABL1* to *ABL1* percent (IS standardized). The limit of detection for the clinical assay is ~0.0030% IS. The DCB research sample was obtained using Tasso-M20 within 10 days of the clinical *BCR::ABL1* transcript measurement. After placing a heating pad for 2–5 min, Tasso-M20 was applied to the deltoid muscle via adhesive. Pushing on the device’s button creates a vacuum and deploys a lancet to draw capillary blood. The Tasso-M20 device collects 70 μl of capillary blood in 4 volumetric tips, which then dry. To simulate the time for shipment and transportation expected in real-world applications, Tasso-M20 samples were sent to the laboratory, maintained at room temperature, and processed within two weeks of collection (mean, 8.8 days and range, 6–13 days). Briefly, volumetric tips were placed in Cepheid lysis buffer diluted with molecular water. Proteinase K was added, and the sample was incubated for one hour. The lysate was then transferred to a new tube, ethanol was added, and this mix was loaded into the Cepheid Xpert® BCR-ABL Ultra cartridge and run on a GeneXpert machine. *BCR::ABL1*/*ABL1* IS percent ratios are reported (full protocol in Supplementary Materials).

Patient samples were obtained between October 1, 2020 and October 31, 2021. Median age was 51.5 years (range, 28–83 years) and 55% were female. The ethnic and racial distribution of patients reflected regional  demographics and included 5% American Indian or Alaska Native, 15% Asian, 5% Black or African American, and 5% Hispanic or Latino individuals. Before we began utilizing a heating pad routinely, the device was deployed a 2nd time due to lack of capillary blood flow in 2 patients. The capillary blood draw using Tasso-M20 was well tolerated and associated with less pain than traditional venipuncture. Using a pain scale of 1 to 10, 19 of 20 patients rated pain as < 1.5. Clinical *BCR::ABL1* transcript levels in venous blood ranged from undetectable to 27.98%, over a 4-log_10_ range. Most patients enrolled had *BCR::ABL1* transcripts *≤* 1%, were in MMR, or had *BCR::ABL1 ≤* 0.01% (deep molecular responses required for TKI discontinuation in eligible patients). Approximately 381,990 cells (mean, calculated for 70 μL of blood) were tested (range, 155,400–714,000). Individual patient results are shown in Table 1. As shown in Fig. 1b, there was a strong correlation between the *BCR::ABL1* transcripts in capillary vs. venous blood, (*r* = 0.96 between the two log_10_ transformed measures). Figure 1c displays the bias as a function of the average log_10_ (% IS + 1) with a Bland-Altman plot, where the bias is the difference between the log_10_ transformed values of the venous and capillary samples. For visualization purposes, we represent values below the detection limit as 0.0001. Minimal bias was identified. By DCB measurement 15 patients were reported to have achieved MMR. Among these patients, 12 had achieved MMR by standard of care venous blood measurement. Venous (and capillary) *BCR::ABL1* transcripts for the 3 discordant patients were as follows: 0.13% (vs. 0.016% by DCB), 0.36% (vs. 0.05%), 0.12% (vs. 0.024%). All 5 patients without MMR by DCB testing were identified concordantly in venous testing. Venous *BCR::ABL1* measurements below 0.09% were not reliably detected by DCB measurements.

**Table 1:** Capillary dried blood (Tasso-M20) and venous blood *BCR::ABL1* results in all patients.

Patient	Age (years)	Gender	WBC (10^3^ per μl)	Venous (*BCR::ABL1/ABL1*, %)	Capillary (*BCR::ABL1/ABL1*, %)
**1**	72	M	5.98	0.0047	Not detected
**2**	31	M	7.16	0.13	0.016
**3**	31	F	4.72	0.02	0.029
**4**	63	M	3.18	0.09	0.05*
**5**	51	F	3.76	2.01	0.14
**6**	42	F	6.15	0.021	Not detected
**7**	78	F	6.52	0.36	0.05
**8**	37	F	4.56	0.067	Not detected
**9**	62	F	5.58	0.099	0.045
**10**	52	M	2.35	27.98	12.23
**11**	83	M	4.63	0.034	0.1
**12**	67	F	3.66	Not detected	Not detected
**13**	51	M	10.2	0.022	Not detected
**14**	44	F	4.95	0.014	Not detected
**15**	63	M	7.52	0.23	0.36
**16**	58	F	4.4	0.12	0.024
**17**	37	F	6.75	0.021	0.021
**18**	67	F	8.7	1	0.46
**19**	30	M	2.22	0.065	Not detected*
**20**	28	M	6.15	1.45	0.92

WBC is white blood cell count, M is male, F is female. *ABL1 cycle times (Ct) for #4 and #19 were 19.2 and 18.1, respectively. %*BCR::ABL1* has been calculated manually for sample #4. No amplification for *BCR::ABL1* was observed for #19.

*BCR::ABL1* transcript monitoring was performed using Xpert® BCR-ABL Ultra (Cepheid).

Although earlier efforts have used DBS for therapeutic drug monitoring in CML, we are the first to measure and compare DCB and venous blood *BCR::ABL1* transcripts [11–13]. DCB *BCR::ABL1* transcript monitoring has the potential to improve CML molecular monitoring adherence by allowing local patient-initiated sample collection using regular mail. In its current form, the device and assay provide a strategy to diagnose CML and monitor for treatment responses associated with long-term survival and loss of response that prompts a change in treatment in geographic locations where access to conventional RT-PCR testing is limited by distance, infrastructure, or resource availability. Given the effectiveness of CML therapy, enhanced monitoring access could be a lifesaving intervention. Loss of MMR is also the threshold at which TKI therapy is typically restarted in patients who have discontinued therapy, an intervention that can mitigate chronic side effects, prevent long-term toxicities, improve quality of life, and limit costs to patients and health care systems. DCB provides excellent sample stability and is a cost-effective home diagnostic. However, due to the technical challenges of sampling larger blood volumes, one compromise is limit of detection driven by the smaller numbers of cells investigated. Loss of MMR should be detected by the assay, but the assay would not be appropriate for treatment-free remission  assessment. To improve assay sensitivity, we are modifying the existing Tasso-M20 blood collection cartridges to support a larger capillary blood volume collection. Lastly, the technology could be very useful to continue monitoring patients in future pandemics, where home monitoring would limit the exposure risk that accompanies visits to the clinic. Indeed, we could envision a future where CML patients will be able to summon the collection device by phone (or even drone!), acquire the sample, and send it to a lab safely and reliably.

### Supplementary information

Supplemental material

## Data Availability

The datasets generated and analyzed during the current study are available from the corresponding author upon request.
