# Supplementary material for: Point-of-care BCR::ABL1 transcript monitoring using capillary dried blood in chronic myeloid leukemia patients
Source: Leukemia. 2024 Jun 15;38(8):1822–4. doi: 10.1038/s41375-024-02285-9 (PMC11286517; doi:10.1038/s41375-024-02285-9)
Supplement: Supplementary file 1 — Supplemental material [file 41375_2024_2285_MOESM1_ESM.pdf]

Supplemental Materials

Tasso dried capillary blood sample preparation for *BCR::ABL1* detection using Cepheid Xpert BCR-ABL Ultra kit.

Materials:

-GeneXpert system (Cepheid, California)

-Xpert® BCR-ABL Ultra (Cepheid, California)

-5ml centrifuge tubes

-ThermoMixer C (Eppendorf, Germany)

-100% Ethanol

-P1000, and P200 pipettes and tips.

Methods:

1. Prepare the "working lysis solution" (WLS) by adding 1 part of molecular water to 4 parts of lysis buffer (Xpert® BCR-ABL Ultra).
2. Transfer the four dried Tasso volumetric tips to a 5mL Eppendorf tube.
3. Add 2.75mLs of the WLS and 100 µL of Proteinase K (Xpert® BCR-ABL Ultra).
4. Incubate the tube for 1 hour in a Themomixer at 65 °C, 500rpm.
5. After the incubation, transfer the lysis to a clean tube.
6. Add 2mLs of 100% Ethanol to the sample.
7. Mix by vortexing for 10 seconds.
8. Load the sample into the sample chamber of the Cepheid cartridge.
9. Load the Wash Reagent (Xpert® BCR-ABL Ultra) according to Cepheid instructions.
10. Load cartridge on the GeneXpert instrument.
